# Congenital Herpes Simplex Virus: A Histopathological View of the Placenta

**DOI:** 10.7759/cureus.29101

**Published:** 2022-09-13

**Authors:** Theodora-Eleftheria Deftereou, Anna Trypidi, Christina Angelika Alexiadi, Paschalis Theotokis, Maria Eleni Manthou, Soultana Meditskou, Maria Simopoulou, Maria Lambropoulou

**Affiliations:** 1 Laboratory of Histology and Embryology, Democritus University of Thrace, Alexandroupolis, GRC; 2 Department of Physiology, National and Kapodistrian University of Athens, Athens, GRC; 3 Laboratory of Histology and Embryology, School of Medicine, Democritus University of Thrace, Alexandroupolis, GRC; 4 Laboratory of Histology and Embryology, School of Medicine, Faculty of Health Sciences, Aristotle University of Thessaloniki, Thessaloniki, GRC; 5 Laboratory of Histology and Embryology, Aristotle University of Thessaloniki, Thessaloniki, GRC

**Keywords:** placenta, congenital infection, placental histopathology, pregnancy, hsv

## Abstract

Congenital Herpes simplex virus (HSV) infection is considered a common pregnancy pathology that is not always easy to diagnose. This study aimed to present the spectrum of placental histopathological lesions in pregnancies complicated by HSV infection. MEDLINE and Google Scholar databases were searched using the keywords "HSV" and "placental histopathology" up to June 20, 2022. Study inclusion required presenting placental histopathological anomalies in pregnant women diagnosed with HSV infection antenatally, during labor, or postnatally. Herein, we briefly present placental pathogenesis conditions, which have been correlated with congenital HSV infection, providing clinicians with a short review describing herpetic placental pathology.

## Introduction and background

It is widely acknowledged that specific infections during pregnancy may adversely affect both fetus and mother, inducing congenital disorders, intrauterine growth restriction, prematurity, stillbirth, and spontaneous abortions [1]. Vertical transmission is defined as the transmission of a pathogen from mother to fetus in utero through the hematogenous or the ascending route. This may lead to the disruption of organogenesis, which may be related to congenital anomalies in every major organ system [1,2]. Additionally, infection of the fetus through the genital tract during birth may increase the rate of neonatal morbidity and mortality [3].

The acronym "TORCH" which stands for *Toxoplasma gondii*, other agents, rubella virus, cytomegalovirus (CMV), and herpes simplex virus (HSV) has been proposed to describe the major pathogens, which may be related to the development of congenital disease [1,2]. TORCH pathogens can enter the intra-amniotic space and overcome placental defense mechanisms that protect against vertical microbial transmission [1-3].

HSV is a double-stranded DNA virus and a member of the *Herpesviridae *family [4], responsible for oral and genital herpes [5]. As a neurotropic virus, it is detected in the dorsal root ganglion, following primary infection [1,6]. The presence of the G1 and G2 glycoproteins in the lipid bilayer envelope can serve as markers that determine the viral serotype, HSV-1 and HSV-2, respectively [7].

It has been estimated that over 13% of the population within the reproductive age has been infected with HSV-2, a phenomenon that establishes HSV-2 as the most common sexually transmitted infection [8,9]. Thus, the risk of fetal or neonatal transmission is increased as numerous women of reproductive age are considered to be infected [7].

HSV could be transmitted to the fetus through the transplacental or the ascending route of acquisition from the vagina or cervix, even when the amniotic membranes remain intact [10,11]. Despite that, the intrapartum transmission of HSV through contact with a virus-shedding lesion in the genital tract tends to be the most commonly detected transmission route [12]. Maternal-fetal transmission may occur in any of the three pregnancy trimesters [13].

The teratogenic effect of HSV on an embryo may provoke a variety of clinical outcomes, namely, ventriculomegaly, microcephaly, intracerebral calcifications, limb dysplasia [1], eye defects [4], and fetal loss [11,14]. As previously reported, primary infection in late pregnancy increases the risk of vertical transmission [13]. The increased rates of fetal loss and the neurodevelopmental disorders regarding fetal neurons and neuronal precursors following HSV infection are associated with poor prognosis [13,15].

As evidenced by the literature, there are inconclusive results concerning the mechanism responsible for fetus infection by HSV. It has been reported that the expression of HSV entry mediators in syncytiotrophoblast (SYN), namely HveA, HveB, and HveC, provides resistance to viral infection. The inflammation-mediated breakage of the SYN layer allows the virus to reach fetal circulation [1,16]. Furthermore, it has been reported that HSV may be detected in the dorsal root ganglia following the primary infection [17]. Thus, potentially, a transneural migration of HSV to the endometrium may result in uterus transmission during pregnancy [17]. In contrast, the expression of all entry mediators at the extravillous cytotrophoblast (EVT) renders it sensitive to HSV transmission [9,18]. Moreover, the infection of endothelial cells in maternal microvasculature may also induce EVT infection [1,16].

During pregnancy, most of the infected women are asymptomatic or present with nonspecific symptoms. The absence of clinical characteristics could result in a late diagnosis of congenital or neonatal HSV infection, increasing both morbidity and mortality [11,17]. The placenta may be macroscopically normal [19], and its histopathological manifestations may be nonspecific for the HSV infection. Since the placental pathological examination could constitute a strong indication of the viral presence, the current study aims to explore the effects of congenital HSV infection on the placenta’s histopathological profile.

## Review

Placenta histopathological findings

Herpetic placentitis constitutes a microscopic feature in the placental pathological examination, indicating hematogenous transplacental HSV transmission. It is characterized by the co-existence of herpetic villitis, chronic intervillositis, and villous necrosis. Particularly, lymphoplasmacytes may penetrate villi [20], while the extensive presence of histiocytes in intervillous space [21] and areas with villous necrosis and granulomatous reaction may be further identified. Moreover, the literature indicates that necrotic Hofbauer cells and syncytiotrophoblast may also be detected [20,22,23]. Additionally, trophoblastic multinucleation may constitute another finding [21], and the characteristic viral inclusion bodies, also known as HSV Cowdry type B, are frequently identified [10]. Due to the degeneration of HSV-infected cells’ nucleus, Cowdry type B may appear in the amniotic epithelium, trophoblast, chorion, and decidual cells like eosinophilic ground-glass areas in the nucleus with margination of nuclear chromatin. All these findings are considered indicative of HSV infection [20,22-25].

Chronic placental inflammation (CPI) constitutes a common histopathologic finding in infections like herpesvirus (HSV-1 and HSV-2) with high maternal-fetal rate transmission. CPI is characterized by the presence of lymphocytic, histiocytic, and plasmacytic infiltration in the villous tree, similar to villitis, as well as in extraplacental chorioamniotic membranes, chorionic plate, and basal plate of the placenta [26,27]. It should be noted that the aforementioned placental entities may be regularly observed as separate findings.

Chronic chorioamnionitis constitutes an ascending route of HSV infection, in which amniotic multinucleation, degeneration, and necrosis are described as common histopathological findings. Furthermore, these multinucleated residual cells may be detected in the superficial chorion, while the fetal chorion’s role is considered crucial compared to the amnion. Fetal surface and free membranes may be infiltrated by lymphoplasmacytes and less often by histiocytes, whereas the absence of villitis is not atypical [10]. Plasma cells are sparsely observed in chronic chorioamnionitis, while their presence indicates HSV, CMV, or syphilis infection [20,28-31].

Necrotizing chorioamnionitis, also known as stage 3 of maternal inflammatory response in ascending intrauterine infection, is an acute inflammation of the chorioamniotic membranes [28]. Necrotizing chorioamnionitis is accompanied by amnion epithelial necrosis and karyorrhexis of neutrophils. Moreover, thickening and hypereosinophilia may be observed in the amniotic basement membrane [27,32]. Additionally, subamniotic true “blisters” and plasma cells may also be detected [19]. The excessive necrosis of amnion without any evidence of inflammation may be considered as another finding, which has been described as “cloudy membranes” and has been associated with herpetic infection [22,33-36].

**Figure 1:**
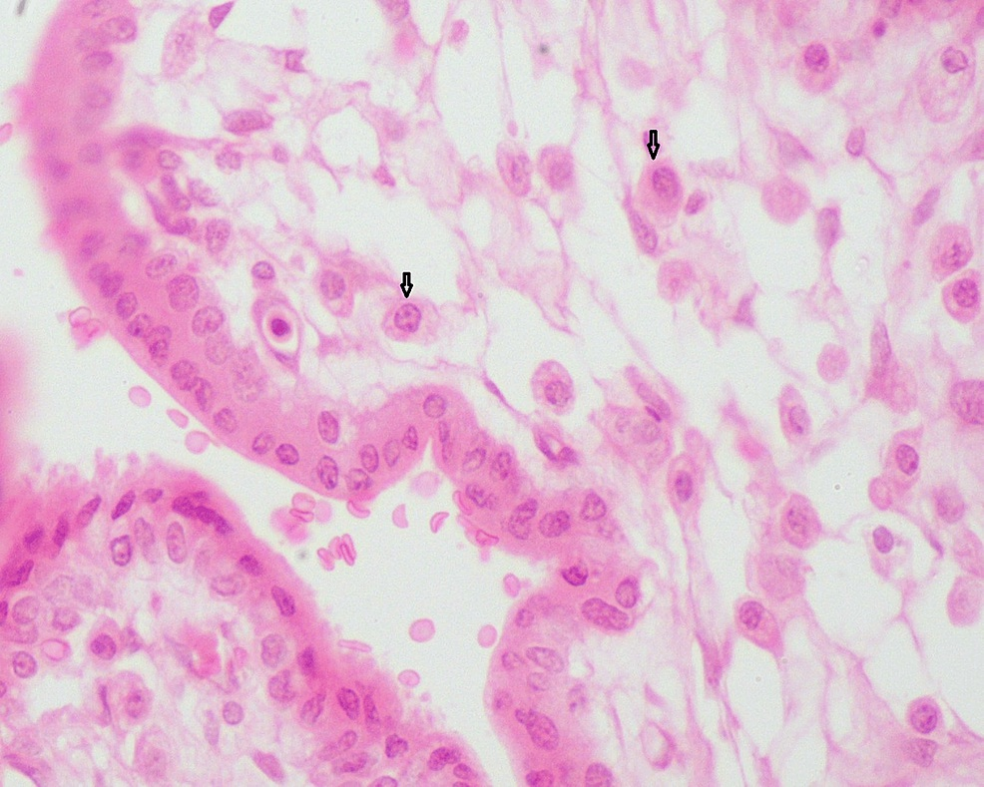
Representative sample of villitis with the presence of plasma cells (indicated by arrows). Hematoxylin/eosin-stained section of placenta in congenital HSV infection (magnification x 400). HSV: Herpes simplex virus. Source: The sample was obtained from the Laboratory of Histology-Embryology Archive, Medical School, Democritus University of Thrace, Greece.

Chronic villitis (CV) refers to the detection of maternal lymphocytes, histiocytes, and, in some cases, plasma cells in placental villi and has been associated with congenital viral infection [14,25], as shown in Figure 1.

In this case, chorionic villi appear hypovascular, without distinct borders [20]. Diagnosis of CV entails the maternal and fetal investigation for viral infection, including HSV [37-40]. In the case of localization of villitis only on the maternal floor, chorionic villi are defined as basal CV. Identification of severe plasma cell infiltration of chorionic villi may constitute an indication of congenital HSV infection, and further investigation is required [20]. Additionally, CV accompanied by multinucleated giant cells may be linked to HSV, varicella, or toxoplasmosis [41].

Villitis of unknown etiology (VUE) is defined as the lymphohistiocytic infiltration of chorionic villi in the absence of a specific infectious pathogen [28]. It is considered a common lesion, especially in full-term placentas [42]. Grossly, the placenta of VUE may be described as stiff and, if villitis is extensive, the villous tree tends to be molted. Microscopically, the villous tree seems to be intensely infiltrated by neutrophils and granulomas, mainly in the basal villi on the maternal floor. Furthermore, ischemia and infraction may be encountered as secondary findings [19,42]. This pattern requires a detailed investigation for a possible congenital infection rather than an isolated clinical incident. Since VUE seems to have a high recurrence rate, HSV transmission may be excluded in these cases [43]. The presence of plasma cells and viral inclusions in VUE cases may indicate congenital HSV [20].

Villous necrosis can be presented in a scattered foci pattern and co-exist with decidual necrosis. It has been reported that bland patchy villous necrosis can indicate an ascending herpetic infection, especially if necrotizing deciduitis or funisitis is also detected. An individual finding can be considered nonspecific but can also be the only evidence for a congenital infection [22,44].

**Figure 2:**
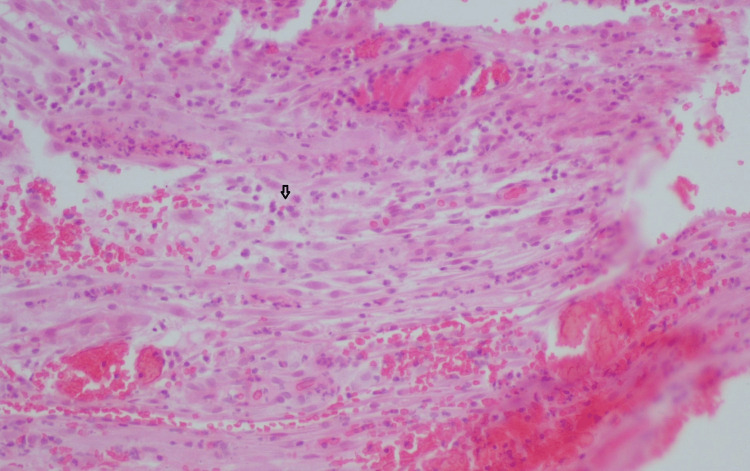
Representative sample of deciduitis. The infiltration is mainly composed of round cells (indicated by the arrow). Hematoxylin/eosin-stained section of placenta in congenital HSV infection (magnification x 200). HSV: Herpes simplex virus. Source: The sample was obtained from the Laboratory of Histology-Embryology Archive, Medical School, Democritus University of Thrace, Greece.

Chronic deciduitis is designated as the focal or diffused detection of lymphocytes and plasma cells in decidua basalis or membranous decidua [20], as presented in Figure 2.

As a chronic inflammatory condition, chronic deciduitis requires maternal and fetal screening for hematogenous infections, including HSV [37,45]. Intervillositis, as a distinct finding in the placental examination, is based on the detection of a characteristic pattern comprising mostly of histiocytic infiltration of intervillous space [46]. Although the underlying etiology remains unclear, the presence of maternal macrophages and the increased incidence of the infection in women with autoimmune disease may provide an insight into the underlying pathophysiological mechanisms, nonetheless. Despite that, chronic intervillositis has been linked to inappropriate activation of the immune response to the semi-allogeneic fetus [46]. Interestingly, intervillositis has also been reported in the placentas of women infected with malaria and/or acute CMV infection [47]. The co-occurrence of chronic intervillositis and malaria has raised questions that an underlying infection could potentially play a role in the onset of chronic intervillositis [47]. As previously documented, infectious intervillositis with a polymorphic infiltrate containing neutrophils and leukocytes indicates acute inflammation [47]. Even not often, among the infectious agents, HSV should be included [32,48]. Considering the high rates of recurrence of intervillositis [28,49], HSV seems to be encompassed in differential diagnosis [19].

Massive perivillous fibrinoid deposition (MPFD) is an extremely rare placenta condition characterized by abnormally extensive fibrinoid deposition in the placental villous parenchyma [50]. Chorionic villi seem to be sclerotic and encased by the extensive deposition of eosinophilic fibrinoid material within the intervillous space. It is characterized by a high rate of recurrence and has also been correlated with autoimmune diseases. Hitherto, the underlying etiology of MPFD remains unclear. Autoimmune disease, infection, toxic reagents, abnormal host-placental interactions, coagulation disorders, and genetic conditions have been proposed as triggering factors for MPFD [50-52]. Regarding infections, coxsackieviruses, syphilis, CMV, syndrome coronavirus 2 (SARS-CoV-2), and congenital HSV infection have been associated with MPFD pathogenesis [53,54]. It has also been reported that this condition represents a final common pathway for a number of miscellaneous disorders culminating in chorionic villus injuries associated with intervillous circulation stasis [50].

Chronic chorionic vasculitis (CCV) is an uncommon placental lesion, rarely affecting multiple vessels. Its diagnosis includes the detection of fetal lymphocytes and eosinophils in the chorionic vessel wall. It constitutes the first stage of fetal inflammatory response and is often associated with CV. In some cases, a thrombus may also be observed as a response to inflammation [45]. It has been reported that transplacental or ascending HSV infection may induce CCV [28,41,55].

Chronic funisitis is also an atypically detected lesion. It is defined primarily as lymphocytic infiltration of the umbilical cord due to the establishment of a chronic in-utero viral infection. It has been speculated that HSV may induce chronic funisitis [10,56], while the presence of plasma cells in funisitis requires the exclusion of a herpetic infection [11,20].

Necrotizing funisitis (NF) is characterized by severe umbilical cord inflammation, which could be both grossly and microscopically detected. Under the microscope, NF is depicted as a discontinuous pattern comprising areas with neutrophilic or lymphocytic infiltration and areas enriched with necrotic debris. Additionally, calcium deposits can be frequently detected. Despite that NF is predominantly associated with congenital syphilis, this lesion has been also linked with ascending congenital HSV infection [28,57,58].

Villous agglutination constitutes a nonspecific histopathological finding in the placental examination. It has been described as clusters of adherent distal villi agglutinated by fibrin and/or bridging syncytial knots, which are not separated by maternal blood space [20,59]. It was initially associated with congenital rubella [60] and recently attributed to SARS-CoV-2 [61]. It should be noted that congenital HSV infection cases may also be presented as villi agglutinated without any evidence of villous or membranes’ inflammation [9].

**Figure 3:**
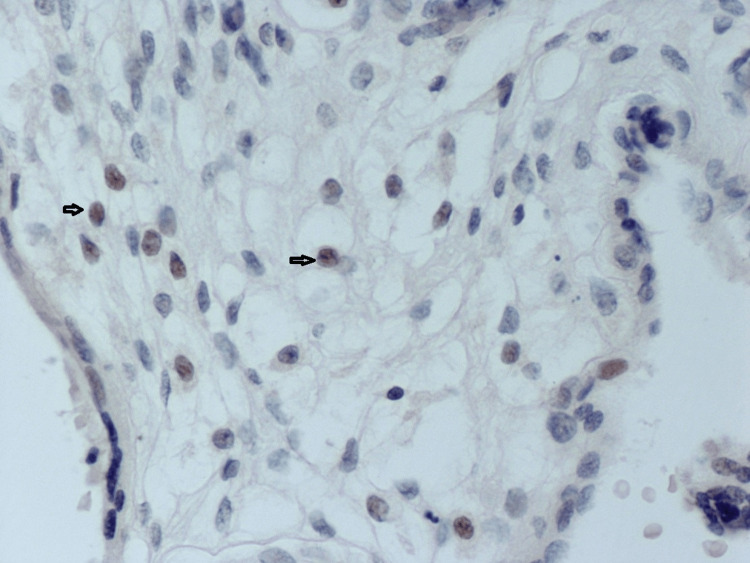
Immunohistochemical staining for HSV-1 in placental tissue complicated with congenital HSV infection (magnification x 200) (Abcam, rabbit polyclonal). Arrows indicate the positively stained cells. HSV: Herpes simplex virus. Source: All placental samples were obtained from the Laboratory of Histology-Embryology Archive, Medical School, Democritus University of Thrace, Greece.

All the findings above should raise suspicions of an HSV infection, which can be confirmed via immunohistochemical staining as presented in Figure 3.

In Table 1, the main histopathological findings associated with congenital HSV infection are briefly presented.

**Table 1:** Summary of placental histopathological findings associated with congenital HSV infection and the possible mode of transmission L: Lymphocytes; H: Histiocytes; P: Plasma cells; E: Eosinophils; HSV: Herpes simplex virus.

Histopathological findings	L	H	P	Ε	Necrosis	Cowdry type B bodies	Thrombus	Fibrin deposition	Transplacental transmission	Ascending transmission
Chorioamnionitis	+	+	+		+	+				+
Villitis	+	+	+		+	+			+	
Intervillositis	+	+						+	+	
Deciduitis	+		+			+				+
Funisitis	+		+		+				-	+
Vasculitis	+			+			+		+	

In Table 2, the reported cases concerning the histopathological lesions of the placenta due to HSV infection in pregnancy are summarized.

**Table 2:** Summary of the clinical and pathological features of the reported cases diagnosed with congenital HSV infection HSV: Herpes simplex virus.

Authors, year	Gestational age (weeks)	Placenta weight (g)/dimensions	Pathology examination
Edwards et al. [10], 2015	29^4/7^	Not presented	Chronic chorioamnionitis
			Degeneration of amnion epithelium
			HSV Cowdry type B intranuclear inclusions
			Chronic fetal inflammatory reaction on umbilical cord
Smith et al. [11], 2020	25^1/7^	Not presented	Subacute necrotizing inflammation with stromal cell necrosis
			Detection of plasma cells
Pfister et al. [24], 2013	27^1/7^	Not presented	Viral inclusions in the amniotic epithelium
			Acute and chronic funisitis
			Acute chorioamnionitis
Chatterjee et al. [29], 2001	27	229.8 g/, 16.3 x 14.7 x 1.2 cm	Necrotic abscess in intervillous spaces
			Intense neutrophilic infiltration
			Karryorrhexis
			Degeneration
			Chronic inflammation of the membranes (predominally lymphocytes)
Kinoshita et al. [31], 2021	29	Not presented	Chronic chorioamnionitis (stage 2): leukocyte infiltration of chorionic membrane
Vasileiadis et al. [33], 2003	27^4/7^	Not presented	Desquamation of the amnion with areas of necrotizing inflammation
Barefoot et al. [34], 2002	31^2/7^	Not presented	Inflammation and necrosis of the fetal membranes
			Chronic funisitis
Amidzic et al. [39], 2017	31	15 cm in the greatest dimension	Mild chronic villitis
			Mononuclear inflammatory infiltration in chorionic villi stroma
Bedolla and Stanek [44], 2004	15	120 g	Villous necrosis
			Decidual necrosis
Bougioukas et al. [55], 2021	26^2/7^	Not presented	Chorioamnionitis
			Funisitis
			Focal chronic chorionic plate vasculitis
			Necrotic stromal cells
Demeulemeester et al. [56], 2015	28	Not presented	Acute chorioamnionitis
			Acute vasculitis
			Acute and chronic funisitis
Wang et al. [58], 2021	31	Not presented	Acute chorioamnionitis
			Necrotizing funisitis

## Conclusions

In this narrative review of placental lesions caused by congenital HSV infection, we have summarized the common and rare histopathological findings associated with HSV infection during pregnancy. Reviews relative to HSV congenital infection and case reports were used. The co-existence of villitis, intervillositis, necrosis, and viral inclusion bodies defined as herpetic placentitis is indicative of transplacental transmission but is not often observed. In cases of villitis, VUE, and intervillositis, the detection of plasma cells should guide the differential diagnosis in a viral infection, including HSV. Chorioamnionitis with or without necrotic lesions and funisitis are less often but can also associate with HSV infection. Additionally, findings such as Cowdry type B on fetal tissues, fibrinoid deposition, and villous agglutination should be investigated as they could be part of an atypical manifestation of HSV infection.

The diagnosis of congenital HSV infection is challenging. Data support that clinical findings of HSV infection may not provide an accurate and precise diagnosis. As herpetic transmission rates increase, congenital HSV infection tends to be a more commonly seen phenomenon, attributed to the fact that physicians lack awareness of the true HSV transmission extent. Determining the route of transmission and distinguishing between hematogenous placental or ascending acquisition of HSV may play a key role in the prognosis of the pregnancy outcome. Placental histopathological examination is of utmost importance as it often dictates the subsequent diagnosis and management of HSV. Further investigation into congenital HSV infection may clarify the placental involvement in the pregnancy outcome.
